# Supra-Orbital Neuralgia Cured by Nerve-stretching

**Published:** 1880-04-20

**Authors:** 


**Supra-Orbital Neuralgia Cured by Nerve-stretching.**—Dr. Kocher relates, in the *Correspondenceblatt fur Schweizer Aerzte*, the case of a man aged 32, who had for seventeen years suffered from neuralgia of the right supra-orbital nerve. The attacks, at first rare, afterwards became more frequent, until at last there were only brief intervals of freedom from pain. All the ordinary therapeutic measures had been tried for years without success.

Dr. Kocher laid bare the nerve and three of its branches by an incision along the upper border of the orbit, and stretched it forcibly by means of an aneurism-needle passed under it. The healing of the wound was attended with abundant suppuration. From the moment of the operation, the patient was free from pain, and the neighborhood of the supra-orbital nerve was anæsthetic. The patient was last seen three months after the operation; he had had no return of the pain; sensation was diminished over a space ten *centimetres* in extent, but was otherwise perfectly restored.

After neurectomy, paroxysms of pain are usually observed during the first few days after the operation. As these were absent in the present case, Dr. Kocher concludes that the lesion of the nerve is less when the nerve is stretched than when it is divided. The value of nerve-stretching as a substitute for excision will be greater in neuralgia of the second and third divisions of the fifth nerve, as here a much smaller wound will suffice.—*British Med. Journal.*

